# Assessment of Depressive Symptoms in Patients with Type 1 and Type 2 Diabetes Mellitus and with Diagnosed Prediabetes in Poland: A Cross-Sectional Study

**DOI:** 10.3390/jcm14062093

**Published:** 2025-03-19

**Authors:** Mariola Mroz, Dorota Sadowska, Mateusz Zarychta, Grazyna Iwanowicz-Palus, Adam Kretowski, Mateusz Cybulski

**Affiliations:** 1 Department of Development in Midwifery, Faculty of Health Sciences, Medical University of Lublin, 20-081 Lublin, Poland; 2 Department of Endocrinology, Diabetology and Internal Medicine, Medical University of Bialystok, 15-276 Bialystok, Poland; 3 Faculty of Technical Physics, Information Technology and Applied Mathematics, Lodz University of Technology, 93-005 Lodz, Poland; 4 Department of Integrated Medical Care, Faculty of Health Sciences, Medical University of Bialystok, 15-096 Bialystok, Poland

**Keywords:** Beck Depression Inventory (BDI), diabetes, depression, mood disorders, prediabetes, quality of life (QoL)

## Abstract

**Background/Objectives**: Diabetes mellitus is one of the most common chronic diseases worldwide. In addition to short-term and long-term complications, diabetes has a detrimental effect on the patients’ mood. The main psychiatric disorder occurring among diabetic patients is depression. The rates of depression in the developed and developing countries are 15% and 11%, respectively. This study aimed to determine the prevalence of depressive symptoms among patients with type 1 and type 2 diabetes and prediabetes in Poland using the example of the Podlaskie Province and taking into account selected sociodemographic variables. **Methods**: A total of 874 patients participated in the study, including 448 women (55.8%) and 386 men (44.2%). The study was conducted from July 2022 to July 2023 among the participants of the “Zatrzymaj cukrzycę! Polski Rejestr Diabetologiczny PolRed” (“Stop Diabetes! Polish Diabetes Registry (PolRed)”) project or those hospitalised in the Department of Endocrinology, Diabetology and Internal Medicine at the University Clinical Hospital in Bialystok. The study used a diagnostic survey method using a survey questionnaire developed by the authors and the Beck Depression Inventory (BDI). **Results**: The highest severity of depressive symptoms according to the Beck Depression Inventory was found in patients with type 2 diabetes (M = 12.18; SD ± 9.48) and the lowest in those with type 1 diabetes (M = 8.11; SD ± 7.55). The assessment of the differences in the severity of depressive symptoms according to the Beck Depression Inventory showed that participants with type 1 diabetes differed statistically significantly (*p* < 0.001) from those with type 2 diabetes and from those in a prediabetic state. In the group of type 2 diabetes (r = 0.336; *p* < 0.001) patients and prediabetic state patients (r = 0.231; *p* < 0.01), there were positive correlations of age with the severity of depressive symptoms. In the group of participants with type 2 diabetes, a statistically significant relationship (*p* < 0.001) was observed between age and the severity of depressive symptoms. **Conclusions**: The prevalence of mood disorders in patients with type 1 and type 2 diabetes and diagnosed prediabetes from the Podlaskie Province depends on the type of hyperglycaemic disorder. The prevalence of depressive symptoms among patients with type 1 and type 2 diabetes and prediabetes is determined by specific socio-demographic factors, including, above all, age and gender. The highest severity of a disturbed emotional state according to the Beck Depression Inventory is found in individuals with type 2 diabetes and the lowest in those with type 1 diabetes.

## 1. Background

Diabetes is one of the most common chronic diseases worldwide [1]. According to the International Diabetes Federation, nearly 537 million people worldwide had diabetes in 2021, including 61 million in Europe. This figure is expected to rise to more than 783 million by 2045 [1]. According to the estimates from the NCD Risk Factor Collaboration (NCD-RisC), as many as 828 million adults (people aged 18 years or older) had diabetes in 2022, an increase of 630 million compared to 1990 [2]. According to the International Diabetes Federation, 2.67 million people in Poland had diabetes in 2021 [1].

Diabetes mellitus is a group of metabolic disorders involving carbohydrate metabolism, in which glucose is inadequately used as an energy source and, at the same time, is overproduced due to abnormal gluconeogenesis and glycogenolysis, leading to hyperglycaemia [3]. Diabetes mellitus is characterised by chronically elevated blood glucose levels due to the insufficient insulin production caused by an autoimmune destruction of pancreatic beta cells (β-cells) and insufficient insulin utilisation by the body [3]. Depending on the pathophysiology, diabetes is classified into four categories, i.e., type 1 diabetes, type 2 diabetes, and other specific types of diabetes and gestational diabetes [3].

In addition to short-term and long-term complications, diabetes has a detrimental effect on the patients’ mood. The main psychiatric disorder that occurs among diabetic patients is depression [4,5]. The rates of depression in the developed and developing countries are 15% and 11%, respectively [6]. Chen et al., in their meta-analysis, showed that the prevalence of depression positively correlated with prediabetes, undiagnosed diabetes, and previously diagnosed diabetes, compared to healthy individuals [7]. Depression often remains undiagnosed [8]. Depression and anxiety are the fourth reason, and diabetes is the eighth reason for disability-adjusted life years (DALYs) in developed countries [9]. Psychosocial factors, including depression, stress, and poor social support, can lead to, or worsen, diabetes [10].

In light of the above, this study aimed to determine the prevalence of depressive symptoms among patients with type 1 and type 2 diabetes and prediabetes in Poland, as exemplified by the Podlaskie Province, taking into account selected sociodemographic variables such as age, gender, place of residence, and BMI value. To achieve the research objectives, the following research question was formulated: what is the prevalence of depressive symptoms, as measured by the Beck Depression Inventory (BDI), in individuals with type 1 diabetes, type 2 diabetes, and prediabetes in the Podlaskie Province of Poland, and how do these symptoms vary by sociodemographic factors? We hypothesised that the prevalence of depressive symptoms varies depending on the type of diabetes diagnosed. The following two specific hypotheses were also proposed: (1) the prevalence of mood disorders in patients with type 1 and type 2 diabetes and those diagnosed with prediabetes is determined by the indicated socio-demographic factors and (2) there are differences in the mood scores of patients with type 1 and type 2 diabetes and those diagnosed with prediabetes.

## 2. Materials and Methods

### 2.1. Study Group

The study included 874 patients, 448 women (55.8%) and 386 men (44.2%), participating in the “Zatrzymaj Cukrzycę! Polski Rejestr Diabetologiczny (PolRed)” (“Stop Diabetes! Polish Diabetes Registry (PolRed)”) project or hospitalised in the Department of Endocrinology, Diabetology and Internal Medicine of the University Clinical Hospital in Bialystok. Participants from the Polish Diabetes Registry (PolRed) project were mainly patients of the Diabetes Clinic operating at the Department, and most of them have been hospitalised in the Department in the past.

A minimum sample size of at least 384 enrolled individuals would have been required to investigate the selected variables in diabetics in Poland (i.e., 2.67 million people with diabetes in Poland in 2021). The sample was calculated by a sample size calculator, based on the reference population, assuming a response proportion of 50%, a 95% confidence level, and a 5% margin of error.

### 2.2. Study Design

The study was conducted between July 2022 and July 2023. Respondents were informed of the anonymity and the voluntary nature of their participation in the study, and that the results obtained would be used for research purposes only. Consent was obtained from patients to participate in the study after discussing the study’s design and purpose with them.

The inclusion criteria for the study were as follows:

- written consent to participate in the study,
- age above 18 years,
- a diagnosis of type 1 diabetes, type 2 diabetes, or prediabetes (prediabetes was diagnosed based on laboratory measurement of fasting blood glucose (FBG)),
- no psycho-physical disorders (persons able to reliably describe their subjective feelings and to fill in the questionnaire form independently).

The exclusion criteria for the study were as follows:

- lack of consent to participate in the study,
- age below 18 years,
- no diagnosis of type 1 diabetes, type 2 diabetes, or prediabetes,
- existing psycho-physical disorders; in particular, the study excluded people currently undergoing treatment for depression or anxiety and people with cognitive impairment.

In the first stage of organising the research work, a preliminary analysis of the literature on the research topic was carried out, based on which the research methodology and concept of the study were developed—it’s aim, main problem, specific problems, and research hypotheses. The scientific method and technique were defined, and appropriate research tools were prepared. Permission to conduct the study was obtained from the Head of the Department of Endocrinology, Diabetology and Internal Medicine of the University Clinical Hospital in Bialystok and the Clinical Research Centre of the Medical University of Bialystok.

A clinical psychologist from the Department of Endocrinology, Diabetology and Internal Medicine at the University Clinical Hospital in Bialystok was involved in the implementation of the study, providing support for the patients participating in the study who were classified as having symptoms of borderline clinical depression or at least symptoms of moderate depression, based on the Beck Depression Inventory.

The success rate of the data obtained was 93.38%. Questionnaires with incomplete data or incorrectly completed sheets were not further analysed.

### 2.3. Measures

The research was carried out via a diagnostic survey using the questionnaire technique and employing a document study using the document analysis technique. Medical records of patients of the Department of Endocrinology, Diabetology and Internal Medicine of the University Clinical Hospital in Bialystok and the Clinical Research Centre of the Medical University of Bialystok were used for the analysis.

The research tool consisted of two components:

- the survey questionnaire developed by the authors, taking into account the characteristics of the respondents (sociodemographic variables),
- the Beck Depression Inventory (BDI)—a self-report scale for diagnosing depression. The questionnaire allows qualitative and quantitative measurement—making a preliminary diagnosis and assessing the level of severity of symptoms. It consists of 21 items relating to the most commonly observed symptoms of depression. The questions are grouped into four categories:
  1. somatic (physical) symptoms,
  2. affective (emotional) symptoms,
  3. cognitive symptoms,
  4. vegetative symptoms (referring to changes in sleep patterns and appetite).

For each question, one of four answers can be selected, which is assigned a value from 0 to 3 points. The person who completes the questionnaire assesses the degree to which they experience the symptoms, using a 4-point rating scale: 0—none, 1—moderate, 2—considerable, 3—very considerable. A response score of 0 indicates that the person has no problems regarding depression, while the last response option ticked, a score of 3, indicates a severe problem related to a particular symptom. The Beck Depression Inventory score is based on the sum of the scores, ranging from 0 to 63. The scores awarded for the answers to the individual questions are added together to give an overall score that indicates the severity of depression.

The different scoring ranges correspond to the following outcomes:

1. score 1–10 points: changes in mood are considered normal,
2. score 11–16 points: mild mood disturbance,
3. score 17–20: borderline clinical depression,
4. score 21–30: moderate depression,
5. score 31–40: severe depression,
6. score of 40 or more: extreme depression.

The Beck Depression Inventory, although widely used to determine the severity of a person’s depression, is not used as the sole basis for diagnosis or treatment for depression. The Beck Depression Questionnaire is often used in research as well as in clinical practice as a tool to help diagnose and monitor the severity of depressive symptoms. The Cronbach’s α coefficient for the BDI ranges from 0.92 to 0.95 [11,12,13,14].

### 2.4. Procedure and Ethical Considerations

The study was conducted following the recommendations and was reviewed and approved by the Bioethics Committee of the Medical University in Bialystok (statute no. APK.002.264.2022 of 23 June 2022). All participants gave written informed consent in accordance with the Declaration of Helsinki.

### 2.5. Statistical Analysis

The collected research material was statistically processed using the statistical package IBM SPSS Statistics (ver. 21) (IBM, Armonk, NY, USA).

Quantitative variables were described by mean, standard deviation, quartiles, measures of symmetry and kurtosis, as well as minimum and maximum values. For qualitative variables, the count and percentage of each category were provided.

Appropriate statistical tests were applied to verify the hypotheses. The normality of data distribution was checked using the Shapiro–Wilk test. When the assumptions for parametric tests (variables measured at the quantitative level of measurement) were met, the independent samples Student’s t-test was used to verify the hypothesis of equality of means of the analysed variable in two populations, and the one-way analysis of variance (ANOVA) for independent groups was used to verify the hypothesis of equality of means of the analysed variable in several populations.

Non-parametric methods were used in the case of relatively large disparities between the compared groups and because of the (ordinal) level of measurement of the variables. A chi-square (χ^2^) test designed to assess the relationship between variables measured on a nominal scale was used to assess the relationship between variables measured on a qualitative scale.

The statistical analyses carried out also used the Tukey test for comparing pairs of means as one of the post hoc tests (multiple comparison procedures), performed after obtaining a significant F-value following an analysis of variance (ANOVA), showing means that differ statistically significantly, taking into account the number of degrees of freedom (df), i.e., the number of independent observational outcomes minus the number of relationships that link these outcomes to each other (the number of degrees of freedom can be equated with the number of independent random variables that influence the outcome).

To determine the correlation between quantitative variables, Pearson’s linear correlation coefficient was used, which is designed to test for a linear relationship between two characteristics as long as the distribution of the analysed characteristics is a normal distribution.

To determine the strength of the correlation, a classification according to J. Guilford was used:

- 0—no correlation,
- 0.0 < |r| ≤ 0.1—slight correlation,
- 0.1 < |r| ≤ 0.3—weak (low) correlation,
- 0.3 < |r| ≤ 0.5—average correlation,
- 0.5 < |r| ≤ 0.7—high correlation,
- 0.7 < |r| ≤ 0.9—very high correlation,
- 0.9 < |r| < 1.0—almost complete correlation,
- |r| = 1—complete correlation.

The results obtained were assumed to be statistically significant at the significance level of 0.05.

## 3. Results

The study comprised 874 subjects from the Podlaskie Province—patients of the Department of Endocrinology, Diabetology and Internal Medicine of the University Clinical Hospital in Bialystok and the Clinical Research Centre of the Medical University in Bialystok—including 124 patients with type 1 diabetes (14.2%), 581 patients with type 2 diabetes (66.5%) and 169 subjects with prediabetes (19.3%). Overall, women (55.8%), people aged 51–70 years (42.2%), residents of provincial cities (50.0%) and those with a BMI indicating obesity (44.2%) predominated in the study group. Detailed data are presented in Table 1.

The assessment of differences in the severity of depression symptom levels according to the Beck Depression Inventory showed that subjects with type 1 diabetes differed statistically significantly (*p* < 0.001) from those with type 2 diabetes and from those with prediabetes (Table 2).

The highest severity of depressive symptoms according to the Beck Depression Inventory was found in patients with type 2 diabetes (M = 12.18; SD ± 9.48) and the lowest in those with type 1 diabetes (M = 8.11; SD ± 7.55).

A statistically significant relationship (*p* < 0.001) was found between health status and severity of depressive symptoms (Table 3). In the group of subjects with type 1 diabetes, there was a higher proportion of subjects with mood changes considered normal (72.6%) compared to subjects with type 2 diabetes (52.3%) and those diagnosed with prediabetes (59.2%). In the group of subjects with type 2 diabetes, there were twice the number of subjects with symptoms of borderline clinical depression (10.5%), as well as symptoms of depression—moderate, severe, extreme (17.4%)—compared to subjects with type 1 diabetes (4.8% showed symptoms of borderline clinical depression, and 9.7% showed symptoms of at least moderate depression).

In the group of subjects with type 2 diabetes (r = 0.336; *p* < 0.001) and prediabetes (r = 0.231; *p* < 0.01), there were positive correlations of age with the severity of depressive symptom levels—the higher the age of the subjects, the higher the severity of depressive symptoms (Table 4).

In the group of subjects with type 1 diabetes, there was no statistically significant relationship (*p* > 0.05) between age and the severity of the level of depressive symptoms. In the group of participants with type 2 diabetes, a statistically significant relationship (*p* < 0.001) was observed between age and the severity of depressive symptoms. In the group of respondents with type 2 diabetes aged up to 50 years, there was the highest proportion (63.4%) of patients whose mood changes were considered normal. This age category also had the lowest proportion (9.7%) of individuals who showed symptoms of at least moderate depression. On the other hand, in the age group over 70 years, the percentage of people whose mood changes were considered normal was found to be the lowest (33.3%). Furthermore, in this age category, almost one in three (29.5%) respondents showed symptoms of at least moderate depression, and one in eight (13.5%) showed symptoms of borderline clinical depression. In the group of respondents with prediabetes, there was a statistically significant relationship (*p* < 0.05) between age and the severity of the level of depressive symptoms. In the group of respondents with prediabetes aged 51 to 70 years, there was the highest percentage (67.2%) of those whose mood changes were considered normal. In contrast, in the age group above 70 years, the percentage of people whose mood changes were considered normal was found to be the lowest (34.5%). Among the oldest respondents, almost one in four (24.1%) showed symptoms of at least moderate depression, and one in ten (10.3%) respondents showed symptoms of borderline clinical depression (Table 5).

In the group of respondents with type 2 diabetes, there was a statistically significant relationship (*p* < 0.001) between gender and the severity of mood disorders. In the group of male respondents with type 2 diabetes, there was the highest proportion (61.8%) of patients whose mood changes were considered normal. In this group of respondents, the proportion of men who showed symptoms of borderline clinical depression (7.1%) or at least moderate depression (13.1%) was also almost twice as low as that of female respondents with type 2 diabetes (symptoms of borderline clinical depression—13.4%, and symptoms of at least moderate depression—21.0%, respectively) (Table 5).

There was no statistically significant relationship (*p* > 0.05) between the place of residence and severity of mood disorders in the type 1 diabetes and type 2 diabetes groups or in the group of patients diagnosed with prediabetes. In each of the study groups—with type 1 diabetes, type 2 diabetes and diagnosed prediabetes—regardless of place of residence, the majority were those with mood disorders considered normal (provincial city: 49.5–70.0%; city/town other than provincial: 52.8–73.6%; rural area: 50.0–81.8%, respectively), and a minority were those with at least moderate depressive symptoms (provincial city: 10.0–20.4%; city/town other than provincial: 8.9–15.0%; rural area: 0.0–10.2%) (Table 5).

There was no statistically significant relationship (*p* > 0.05) between the type of mood disorder and the BMI of the study participants (Table 5).

In the group of subjects with type 1 diabetes, there was no statistically significant relationship (*p* > 0.05) between gender and the severity of depressive symptoms. In the group of subjects with type 2 diabetes, there was a statistically significant relationship (*p* < 0.001) between gender and the severity of depressive symptoms according to the Beck Depression Inventory. Women showed a higher severity of depression symptoms (M = 13.60; SD ± 9.41) than men (M = 10.52; SD ± 9.30). There was a statistically significant relationship (*p* < 0.05) between gender and the severity of depressive symptoms according to the Beck Depression Inventory in the prediabetic group. Women showed higher severity of mood disorders and depression severity (M = 11.01; SD ± 8.32) than men (M = 8.62; SD ± 6.52) (Table 6).

Post hoc tests showed statistically significant differences (*p* < 0.05) in the severity of depressive symptoms according to the Beck Depression Inventory, depending on the place of residence of the total number of respondents. Differences were present between provincial cities and cities/towns other than provincial cities and between provincial cities and rural areas as the place of residence. Residents of provincial cities showed higher Beck Depression Inventory scores (M = 12.09; SD ± 9.38) compared to residents of cities/towns other than provincial (M = 10.46; SD ± 8.71) or residents of rural areas (M = 9.82; SD ± 8.06). Post hoc tests showed no statistically significant differences (*p* > 0.05) in the severity of depressive symptoms according to the Beck Depression Inventory, depending on the place of residence, for type 1 and type 2 diabetes patients and for those diagnosed with prediabetes (Table 7).

There was no statistically significant correlation (*p* > 0.05) between the body mass index (BMI) and mood disorders (severity of depressive symptoms), as determined based on the Beck Depression Inventory, neither for type 1 and type 2 diabetes patients or for those with prediabetes (Table 8).

## 4. Discussion

Research reports indicate that the co-occurrence of many chronic diseases is interrelated with depression. Also, in the case of diabetes, patients are more likely to develop psychosocial problems [15,16].

A review of the literature provides evidence that the prevalence of depression is increased in patients with diabetes, prediabetes, and in patients with undiagnosed diabetes, compared to those with normal glucose metabolism. The prevalence of depression can be even twice as high in patients with type 2 diabetes and three times as high in those with type 1 diabetes, compared to the overall world population [7,16,17]. On the other hand, depression may also increase the risk of developing type 2 diabetes [17,18].

Studies by Rymkiewicz et al. (2022) [19] and Kowalska-Wojtysiak et al. (2020) [20] showed the presence of depressive symptoms in a significant group of patients with type 2 diabetes. Among the type 2 diabetes patients in a study by Namdeo et al. (2023) [21], the rate of depression was 62%, while 38% of those with diabetes had no or minimal depression problems, 30% had mild depression, 23% had moderate depression, and 9% of patients had moderately severe or severe depression. The prevalence of depression, reported by Roy and Lloyd (2012), was more than three times higher in individuals with type 1 diabetes (12%) and almost twice as high in those with type 2 diabetes (19.1%) compared to individuals not suffering from the disease [16].

A meta-analysis of the studies conducted by Anderson et al. (2001) found that the risk of depression among individuals with diabetes was twice that of a comparison group without diabetes (OR = 2.0, 95% CI 1.8–2.2) and did not differ depending on the type of diabetes [22]. Both Anderson et al. (2001) [22] and Zubek et al. (2019) [23] noted no differences between the diabetes groups in their studies in terms of the prevalence of depression.

In the study presented here, 19.6% of respondents showed symptoms of mild mood disorders, while 23.9% showed symptoms of depression, including clinical borderline depression (9.0%), moderate depression (10.9%), severe depression (3.2%) and extreme depression (0.8%). Among patients with type 1 diabetes, depressive symptoms were present in 14.5% of participants, and symptoms of mild mood disorders were present in 12.9% of cases. In patients with type 2 diabetes, symptoms of depression were present in 27.9%, while symptoms of mild mood disorders were present in 19.8%. In contrast, depressive symptoms in respondents with prediabetes were present in 17.2% of individuals, while mild mood changes were found in 23.7%. In terms of the differences between the study groups in the severity of depressive symptoms according to the Beck Depression Inventory, it was shown that the highest severity of emotional disturbance was present in those with type 2 diabetes (M *=* 12.18) and the lowest in those with type 1 diabetes (M = 8.11), (*p* < 0.001).

The relationship between diabetes and depression may result from two strategies of interaction between these disease entities. It has been suggested that depression may precede diabetes because changes in glucose transport mechanisms in counterregulatory hormones and immune-inflammatory responses influence the development of depression. The resulting changes lead to disruptions in glucose metabolism, resulting in insulin resistance and B-lymphocyte dysfunction. The second mechanism of action may result from the impact of the chronic nature of diabetes and its accompanying symptoms and complications on the level of stress experienced by patients, which may induce emotional disturbances and depression [21,24,25,26].

Sociodemographic factors may also influence the occurrence and severity of mood disorders in patients with diabetes. The mental sphere in the results presented was influenced by the age of the respondents. It was shown that the older the respondents, the higher the severity of depressive symptoms among the patients with type 2 diabetes and prediabetes. In the group over 50, the proportion of study participants with type 2 diabetes and depressive symptoms increased from 19.5% to 43%, while the proportion of those with prediabetes and depressive symptoms increased from 13.1% to 34.4%.

A study by Klimek et al. (2018) [27] also indicates that the incidence of depression increases with age. Morbidity by age group in study participants was 0.6% among those in the age group between 36 and 40 years of age, 5.1% among patients aged 51–55 years and 11.4% among those over 61.

The present authors’ own research also corresponds with the results of the analysis by Rymkiewicz et al. (2022) [19], who showed that depression in patients with type 2 diabetes is more common in older age groups. A high incidence of depressive symptoms in patients over 60 was also found by Kowalska-Wojtysiak [20]. In the group of older patients with diabetes, difficulties with the self-management of the illness and the presence of complications and comorbidities may be of significance in this regard.

Research reports indicate a relationship between the prevalence of depression and the gender of the patients with diabetes. Klimek et al. (2018) found a higher incidence of depression in men (6.6%) compared to women (3.5%) [27]. In contrast, a meta-analysis of studies by Anderson et al. (2001) showed that the prevalence of comorbid depression in patients with diabetes was significantly higher in women (28%) than in men (18%) [22]. Similar results were also obtained by Roy and Lloyd (2012) [16].

Analysis of the material collected in the course of this study showed higher severity of depressive symptoms in women with prediabetes (M *=* 11.01) and type 2 diabetes (M *=* 13.60), compared to men (M *=* 8.62 and M = 10.52, respectively). Furthermore, the analysis of the effect of sociodemographic variables on the prevalence of depression showed that residents of provincial cities scored higher on the Beck Depression Inventory (M = 12.09) compared to residents of rural areas (M = 9.82) and cities/towns other than provincial (M = 10.46). In terms of the association between place of residence and severity of mood disorders in the group of subjects with type 1 diabetes and type 2 diabetes and the group of subjects diagnosed with prediabetes, there was no statistically significant relationship (*p* > 0.05).

In a study by Klimek et al. (2018), the prevalence of diabetes among rural and urban residents was comparable. Among those with diabetes, 5.6% lived in cities/towns, while 3.9% of respondents were rural residents. The authors found no relationship between the prevalence of diabetes and place of residence (*p* = 0.16) [27].

The presence of depressive symptoms in patients with diabetes may worsen the prognosis of the disease, increase the frequency of non-adherence to treatment, and affect the quality of life, so it is crucial to know the mutual impact of these conditions [28,29]. The biological and psychosocial factors that influence the co-occurrence of diabetes and depression are complex and multifaceted, and it is therefore very important to treat diabetes comprehensively, taking into account the diagnosis and control of disorders of a psychosocial nature and, in addition, also patients with prediabetes. Psychosocial diagnosis should become a routine part of individual patient assessments. This will enable effective prevention and the implementation of early treatment of psychological problems caused by diabetes. Not only pharmacological therapy but also properly designed health education are important methods of treating diabetes, especially if depression is present. With appropriate diabetes care, it is also possible to actively stimulate the patient’s perceived quality of life. It should be remembered that the treatment of diabetes should be holistic, so that the measures indicated are an integral part of the holistic care of patients with diabetes and diagnosed prediabetes.

### Limitations

The study is not without its limitations. First, it was a cross-sectional study. A cross-sectional study is useful for assessing quality of life at one point in time because it allows for collecting data at a specific point in time, which can be helpful in assessing the overall health, well-being, and perception of quality of life in a given population. However, cross-sectional studies have some important limitations, especially in the context of assessing causality, and, in particular, the inability to determine the direction of causality. In cross-sectional studies, it is not possible to determine what is the cause and what is the effect; the lack of tracking changes over time: a cross-sectional study collects data at one point in time, which limits the ability to analyse how the factors may change or how they influence each other over a long period. Caution is needed when interpreting results; due to the lack of a temporal context, results can be misleading in terms of cause and effect. Relationships can only be described, but conclusions about causes cannot be drawn. In summary, cross-sectional studies are useful for obtaining a picture of the situation at a given point in time, but they do not allow for drawing conclusions about the causes of changes in quality of life. For a deeper understanding of the cause–effect relationships, a longitudinal study would be more appropriate. Second, it was a study based primarily on a self-reported questionnaire. Although the scales used are sensitive instruments, they all focus on subjective symptoms rather than objective clinical criteria, creating a risk of false positives. Third, the study was conducted for a single province and not the whole of Poland, which does not detract from the fact that the group was representative in terms of size in relation to the entire country. Fourth, there was a significant imbalance between the three subgroups of patients studied. Of the total number of patients included in the study, 66.5% were patients with type 2 diabetes, while only 14.2% were patients with type 1 diabetes, and 19.3% were diagnosed with prediabetes. Fifth, the sample was designed as a convenience sample and may therefore be subject to selection bias (e.g., patients from the Polish Diabetes Registry (PolRed) project or hospitalised in the Department may have a more severe course of disease or better access to care). 

## 5. Conclusions

The prevalence of mood disorders in patients with type 1 and type 2 diabetes and prediabetes from the Podlaskie Province is associated with the type of hyperglycaemic disorder. The prevalence of depressive symptoms among patients with type 1 and type 2 diabetes and prediabetes is associated with specific socio-demographic factors, including, above all, age and gender. The highest severity of the disturbed emotional state according to the Beck Depression Inventory is found in individuals with type 2 diabetes and the lowest in those with type 1 diabetes. Further studies with more balanced groups should be planned and conducted in the future to provide the most reliable data possible on this research topic.

The obtained results regarding the symptoms of depression among diabetics and people with prediabetes are of great importance for patient care and treatment strategies. Depression can hinder the effective management of diabetes, lead to poorer glucose control, and an increased the risk of complications. Therefore, the care for patients with diabetes should be comprehensive, taking into account not only the management of sugar levels, but also monitoring and treatment of possible depressive symptoms. In addition, in the case of the coexistence of both diseases, medical personnel can pay special attention to psychological support, patient education and modification of the approach to treatment, offering more frequent consultations and reminders about treatment. For people with prediabetes and symptoms of depression, early implementation of preventive measures is particularly important. In addition to lifestyle strategies, such as improving the diet and introducing physical activity, psychological support and monitoring the patient’s emotional state can also be helpful. Regular screening for depression in patients with diabetes, as well as in those at an increased risk of developing diabetes (e.g., obesity, lack of physical activity), can help detect emotional problems early and implement appropriate treatment. In addition, doctors, nurses and other health care professionals should be trained to recognise the symptoms of depression and the psychological support they can offer patients. In summary, the prevalence of depression symptoms among people with diabetes and prediabetes underlines the need for a comprehensive approach to patient care. Collaborations between doctors, psychologists, dietitians and other specialists are crucial to provide an effective treatment, that not only focuses on controlling glucose levels, but also on improving the patient’s mental health. This allows for a better quality of life, greater motivation for treatment, and a reduced risk of health complications.

Specific areas for future research on this topic could include, in addition to patient self-assessment, the inclusion of a professionally administered measure (e.g., a structured clinical interview for depression) to confirm BDI results. Other tools such as the Patient Health Questionnaire (PHQ-9) or the Generalized Anxiety Disorder-7 (GAD-7) could be used for this purpose. In addition, other potentially important factors such as the duration of diabetes, the presence of complications, glycaemic control (HbA1c), the level of social support, socioeconomic status or the level of physical activity could be included in the analyses.

## Figures and Tables

**Table 1:** Sociodemographic characteristics of respondents (n = 874).

Variable		Type 1 Diabetes (n = 124; 14.2%)		Type 2 Diabetes (n = 581; 66.5%)		Prediabetes (n = 169; 19.3%)		Total (n = 874; 100%)	
		n	%	n	%	n	%	n	%
Age (years)	below 50	96	77.4	93	16.0	79	46.7	268	30.7
	51–70	27	21.8	281	48.4	61	36.1	369	42.2
	over 70	1	0.8	207	35.6	29	17.2	237	27.1
Gender	female	63	50.8	314	54.0	111	65.7	448	55.8
	male	61	49.2	267	46.0	58	34.3	386	44.2
Place of residence	provincial city	60	48.4	299	51.5	78	46.2	437	50.0
	city/town other than provincial	53	42.7	233	40.1	79	46.7	365	41.8
	rural area	11	8.9	49	8.4	12	7.1	72	8.2
BMI	underweight	5	4.0	4	0.7	1	0.6	10	1.2
	normal	53	42.7	115	20.0	47	28.0	215	24.8
	overweight	37	29.8	171	29.7	51	30.4	259	29.8
	obesity	29	23.4	286	49.7	69	41.1	384	44.2

**Table 2:** Analysis of mean score for mood according to the Beck Depression Inventory depending on the diagnosis of type 1 diabetes, type 2 diabetes and prediabetes status.

The Study Group	M	SD	A	K	Min	Max	Q_1_	Me	Q_3_
Type 1 diabetes (I)	8.11	7.55	1.28	1.25	0.00	36.00	2.25	6.00	11.00
Type 2 (II) diabetes	12.18	9.48	1.05	0.86	0.00	50.00	5.00	10.00	17.50
Prediabetes (III)	10.19	7.81	1.18	1.38	0.00	39.00	4.00	8.00	14.00
	F 25.546		df 2		*p* < 0.001		Tukey test I–II; I–III		
Study group (total)	11.22	9.03	1.13	1.11	0.00	50.00	4.00	9.00	16.00

Abbreviations: M—arithmetic mean, SD—standard deviation, A—asymmetry, K—kurtosis, Min—minimum, Max—maximum, Q_1_—lower quartile, Me—median, Q_3_—upper quartile, F—analysis of variance (ANOVA), df—number of degrees of freedom.

**Table 3:** Analysis of the severity of mood disorders depending on the diagnosis of type 1 and type 2 diabetes and prediabetic status.

Severity of Mood Disorders	Type 1 Diabetes		Type 2 Diabetes		Prediabetes		Total	
	n	%	n	%	n	%	n	%
Mood changes considered normal	90	72.6	304	52.3	100	59.2	494	56.5
Mild mood disorders	16	12.9	115	19.8	40	23.7	171	19.6
Symptoms of borderline clinical depression	6	4.8	61	10.5	12	7.1	79	9.0
Symptoms of depression (moderate/severe/extreme)	12	9.7	101	17.4	12	10.1	130	14.9
Total	124	100.0	581	100.0	169	100.0	874	100.0
χ^2^ = 23.801; *p* < 0.001								

Abbreviations: *p*—*p*-value, χ^2^—chi-square test.

**Table 4:** Correlation of age with mood disorders (severity of depressive symptoms) of study participants with type 1 and type 2 diabetes and diagnosed prediabetes.

Age								
Beck Depression Inventory	Type 1 Diabetes		Type 2 Diabetes		Prediabetes		Total Study Group	
	r	*p*	r	*p*	r	*p*	r	*p*
	0.083	0.357	0.336 **	<0.001	0.231 *	0.002	0.327 **	<0.001

Abbreviations: r—Pearson’s correlation coefficient, *p*—*p*-value, *—weak (low) correlation, **—average correlation.

**Table 5:** Analysis of the relationship between mood disorders and sociodemographic variables of type 1 and type 2 diabetes patients and those diagnosed with prediabetes.

Variable			Mood Changes Considered Normal		Symptoms of Mild Mood Disorders		Symptoms of Borderline Clinical Depression		Symptoms of (Moderate, Severe, Extreme) Depression		χ^2^
			n	%	n	%	n	%	n	%	
Age (years)	Type 1 diabetes	<50	72	75.0	12	12.5	3	3.1	9	9.4	3.732; *p* = 0.713
		51–70	17	63.0	4	14.8	3	11.1	3	11.1	
		>70	1	100.0	0	0.0	0	0.0	0	0.0	
		total	90	72.6	16	12.9	6	4.8	12	9.7	
	Type 2 diabetes	<50	59	63.4	16	17.2	9	9.7	9	9.7	54.610; *p* < 0.001
		51–70	176	62.6	50	17.8	24	8.5	31	11.0	
		>70	69	33.3	49	23.7	28	13.5	61	29.5	
		total	304	52.3	115	19.8	61	10.5	101	17.4	
	Prediabetes	<50	49	62.0	19	24.1	7	8.9	4	5.1	14.551; *p* = 0.024
		51–70	41	67.2	12	19.7	2	3.3	6	9.8	
		>70	10	34.5	9	31.0	3	10.3	7	24.1	
		total	100	59.2	40	23.7	12	7.1	17	10.1	
Gender	Type 1 diabetes	male	45	73.8	7	11.5	2	3.3	7	11.5	1.218; *p* = 0.749
		female	45	71.4	9	14.3	4	6.3	5	7.9	
		total	90	72.6	16	12.9	6	4.8	12	9.7	
	Type 2 diabetes	male	165	61.8	48	18.0	19	7.1	35	13.1	19.878; *p* < 0.001
		female	139	44.3	67	21.3	42	13.4	66	21.0	
		total	304	52.3	115	19.8	61	10.5	101	17.4	
	Prediabetes	male	39	67.2	14	24.1	2	3.4	3	5.2	4.735; *p* = 0.192
		female	61	55.0	26	23.4	10	9.0	14	12.6	
		total	100	59.2	40	23.7	12	7.1	17	10.1	
Place of residence	Type 1 diabetes	provincial city	42	70.0	8	13.3	4	6.7	6	10.0	3.610; *p* = 0.729
		city/town other than provincial	39	73.6	8	15.1	1	1.9	5	9.4	
		rural area	9	81.8	0	0.0	1	9.1	1	9.1	
		total	90	72.6	16	12.9	6	4.8	12	9.7	
	Type 2 diabetes	provincial city	148	49.5	57	19.1	33	11.0	61	20.4	8.762; *p* = 0.187
		city/town other than provincial	123	52.8	52	22.3	23	9.9	35	15.0	
		rural area	33	67.3	6	12.2	5	10.2	5	10.2	
		total	304	52.3	115	19.8	61	10.5	101	17.4	
	Prediabetes	provincial city	40	51.3	21	26.9	7	9.0	10	12.8	8.728; *p* = 0.189
		city/town other than provincial	54	68.4	15	19.0	3	3.8	7	8.9	
		rural area	6	50.0	4	33.3	2	16.7	0	0.0	
		total	100	59.2	40	23.7	12	7.1	17	10.1	
Assessment of body weight using BMI	Type 1 diabetes	underweight	3	60.0	1	20.0	0	0.0	1	20.0	5.287; *p* = 0.809
		normal	39	73.6	8	15.1	2	3.8	4	7.5	
		overweight	28	75.7	5	13.5	2	5.4	2	5.4	
		obesity	20	69.0	2	6.9	2	6.9	5	17.2	
		total	90	72.6	16	12.9	6	4.8	12	9.7	
	Type 2 diabetes	underweight	1	25.0	0	0.0	1	25.0	2	50.0	13.653; *p* = 0.135
		normal	56	48.7	18	15.7	11	9.6	30	26.1	
		overweight	93	54.4	36	21.1	18	10.5	24	14.0	
		obesity	153	53.5	60	21.0	30	10.5	43	15.0	
		total	303	52.6	114	19.8	60	10.4	99	17.2	
	Prediabetes	underweight	1	100.0	0	0.0	0	0.0	0	0.0	12.505; *p* = 0.186
		normal	28	59.6	6	12.8	7	14.9	6	12.8	
		overweight	30	58.8	17	33.3	1	2.0	3	5.9	
		obesity	40	58.0	17	24.6	4	5.8	8	11.6	
		total	99	58.9	40	23.8	12	7.1	17	10.1	

Abbreviations: *p*—*p*-value, χ^2^—chi-square test.

**Table 6:** Analysis of the relationship between mood scores according to the Beck Depression Inventory and the gender of type 1 and type 2 diabetes patients and those with diagnosed prediabetes.

The Study Group	Gender	Beck Depression Inventory				
		M	SD	t	df	*p*
Type 1 diabetes	male	7.66	7.84	−0.662	122	0.509
	female	8.56	7.28			
Type 2 diabetes	male	10.52	9.30	−3.960	579	<0.001
	female	13.60	9.41			
Prediabetes	male	8.62	6.52	−2.052	142	0.042
	female	11.01	8.32			
Total	male	9.78	8.77	−4.234	872	<0.001
	female	12.36	9.08			

Abbreviations: M—arithmetic mean, SD—standard deviation, t—independent samples Student’s *t*-test, df—number of degrees of freedom, *p*—*p*-value.

**Table 7:** Analysis of the relationship between mood score obtained in the Beck Depression Inventory and the place of residence of type 1 and type 2 diabetes patients in the study and those with a diagnosed prediabetes.

The Study Group	Place of Residence	Beck Depression Inventory					
		M	SD	F	df	*p*	Tukey Test
Type 1 diabetes	Provincial city (I)	8.47	7.31	0.181	2;121	0.835	-
	city/town other than provincial (II)	7.92	7.72				
	rural area (III)	7.09	8.54				
Type 2 diabetes	Provincial city (I)	12.98	9.80	2.780	2;166	0.063	-
	city/town other than provincial (II)	11.61	9.15				
	rural area (III)	10.02	8.56				
Prediabetes	Provincial city (I)	40.42	16.16	2.578	2;168	0.079	-
	city/town other than provincial (II)	11.45	8.44				
	rural area (III)	8.75	7.34				
Total	Provincial city (I)	12.09	9.38	4.219	2;871	0.015	I–II; I–III
	city/town other than provincial (II)	10.46	8.71				
	rural area (III)	9.82	8.06				

Abbreviations: M—arithmetic mean, SD—standard deviation, F—analysis of variance (ANOVA), df—number of degrees of freedom, *p*—*p*-value.

**Table 8:** Correlation of BMI with mood disorders (severity of depressive symptoms) of study participants with type 1 and type 2 diabetes and those diagnosed with prediabetes.

BMI (Body Mass Index)								
Beck Depression Inventory	Type 1 diabetes		Type 2 diabetes		Prediabetes		Total study group	
	r	*p*	r	*p*	r	*p*	r	*p*
	0.020	0.829	−0.024	0.572	0.035	0.654	0.024	0.476

Abbreviations: r—Pearson’s correlation coefficient, *p*—*p*-value.

## Data Availability

The raw data supporting the conclusions of this article will be made available by the authors on request.
